# Hyphenation of microflow chromatography with electrospray ionization mass spectrometry for bioanalytical applications focusing on low molecular weight compounds: A tutorial review

**DOI:** 10.1002/mas.21898

**Published:** 2024-07-01

**Authors:** Sergey Girel, Isabel Meister, Gaetan Glauser, Serge Rudaz

**Affiliations:** ^1^ Institute of Pharmaceutical Sciences of Western Switzerland University of Geneva Geneva Switzerland; ^2^ Swiss Center of Applied Human Toxicology (SCAHT) Basel Switzerland; ^3^ Neuchâtel Platform of Analytical Chemistry University of Neuchâtel Neuchâtel Switzerland

**Keywords:** bioanalysis, electrospray, mass spectrometry, microflow chromatography

## Abstract

Benefits of miniaturized chromatography with various detection modes, such as increased sensitivity, chromatographic efficiency, and speed, were recognized nearly 50 years ago. Over the past two decades, this approach has experienced rapid growth, driven by the emergence of mass spectrometry applications serving ‐omics sciences and the need for analyzing minute volumes of precious samples with ever higher sensitivity. While nanoscale liquid chromatography (flow rates <1 μL/min) has gained widespread recognition in proteomics, the adoption of microscale setups (flow rates ranging from 1 to 100 μL/min) for low molecular weight compound applications, including metabolomics, has been surprisingly slow, despite the inherent advantages of the approach. Highly heterogeneous matrices and chemical structures accompanied by a relative lack of options for both selective sample preparation and user‐friendly equipment are usually reported as major hindrances. To facilitate the wider implementation of microscale analyses, we present here a comprehensive tutorial encompassing important theoretical and practical considerations. We provide fundamental principles in micro‐chromatography and guide the reader through the main elements of a microflow workflow, from LC pumps to ionization devices. Finally, based on both our literature overview and experience, illustrated by some in‐house data, we highlight the critical importance of the ionization source design and its careful optimization to achieve significant sensitivity improvement.

## 1 INTRODUCTION

Electrospray ionization mass spectrometry (ESI‐MS) is widely recognized in modern bioanalytical science as a key tool supporting drug discovery, biomarker research, routine clinical diagnostics and forensic investigations (Gika et al., 2014; Kahl et al., 2019; Olesti et al., 2021; Shipkova & Svinarov, 2016). The robust performance of ESI‐MS enables a highly sensitive and selective detection method for the characterization of thousands of ionized molecules based on their mass‐to‐charge (*m/z*) ratios and fragmentation patterns. Recent advances in automation, instrument design and software development have significantly enhanced the user friendliness of the technique, enabling stable operation by relatively little experienced personnel, for example, in point‐of‐care testing (Fedick et al., 2017). This development is particularly noteworthy as mass spectrometry increasingly plays a pivotal role in personalized medicine approaches, allowing for a comprehensive exploration of a patient's chemical phenotype to deliver customized solutions. The integration of machine learning algorithms further improves the practical utility of the resulting data, transforming it from a mere readout on several biomarkers to a specific disease pattern with higher predictive power (Banerjee, 2020; Clarke, 2016).

Hyphenation of liquid chromatography (LC) adds an orthogonal separation dimension, as the analytes are sorted before the MS inlet according to their mode of interaction with mobile and stationary phases. The resulting multidimensional datasets are further enriched with information on isomeric and isobaric analytes, which are often indistinguishable by their *m/z* ratios and/or fragmentation patterns. A plethora of LC conditions can be found in terms of selectivity, chromatographic resolution, analysis speed and sensitivity (Gika et al., 2014; Harrieder et al., 2022). However, the choice of the LC setup for a particular analytical workflow should be carefully considered as MS becomes a cornerstone in the analytical process due to its costs and functionalities involved. The majority of current LC/ESI‐MS applications use mobile phase flow rates between 200 μL and 1 mL/min with narrow bore analytical columns of internal diameters (ID) between 2.1 and 4.6 mm. These columns are typically connected to pneumatically assisted heated ionization sources present in nearly every MS instrument (Plumb et al., 1999; Van Dongen & Niessen, 2012; Want et al., 2013). Such setup effectively nebulizes the effluent, allowing these widespread platforms to generate reliable data on relatively abundant and/or easy‐to‐ionize analytes, with high throughput (typically 2–10 samples per hour). By contrast, detection of very low abundant analytes (pM or low nM) in complex biological matrices, or those exhibiting lower stability or ionization efficiency, still requires a considerable effort in this setting (Aubry, 2011; Li et al., 2021).

**Figure 1:**
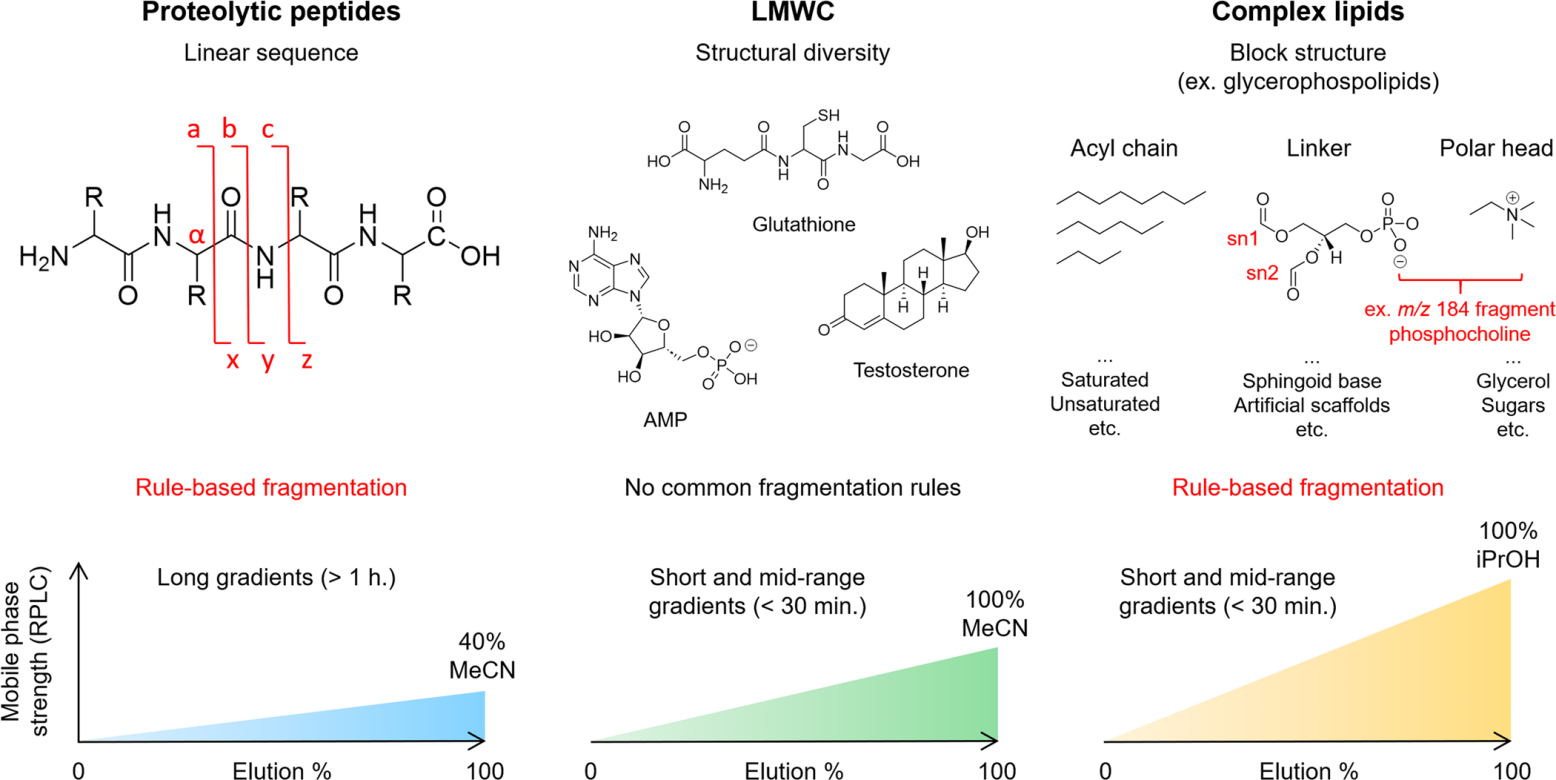
Global comparison between proteolytic peptides, low molecular weight compounds and complex lipids concerning their mass‐spectrometric and reversed‐phase liquid chromatography behaviors. [Color figure can be viewed at wileyonlinelibrary.com]

Miniaturized LC systems with nanoflow chromatography and electrospray regimes have historically dominated certain fields such as bottom‐up proteomics (Ishihama, 2005; Prabhu et al., 2020). With such set ups, the introduction of peptides into the MS inlet at a flow rate of 50–1000 nL/min leads to a remarkable increase in ion generation—up to 500 times more ions than with classic chromatography. Notably, efficient bottom‐up proteomics methods have been recently reported even at higher flow rates of up to 50 μL/min). This approach prioritizes system robustness and throughput, albeit at the expense of sensitivity and proteome coverage (Bian et al., 2020). In this work, we focus on the relatively unexplored field of microflow applications dedicated to low molecular weight compounds (LMWC). LMWC are characterized by a molecular weight below 1500 Da, a high degree of structural heterogeneity and, consequently, an absence of common behaviour in terms of ionization efficiencies and fragmentation patterns. This distinguishes them from proteolytic peptides and lipids (Figure 1).

To measure these more complex and highly heterogeneous analytes in various matrices requires a diversity of sample preparation protocols and chromatographic strategies compared to typical bottom‐up proteomics or lipidomics analyses. The need to concentrate low quantities of analytes from large amounts of sample increases the probability to damage the fragile flow path of microscale systems, such as valves, fittings and electrospray emitters, via precipitation and/or clogging (Chetwynd & David, 2018; Desmet & Eeltink, 2013). Despite these challenges, low flow techniques overall provide great benefits when hyphenated to ESI‐MS detector, and bioanalytical applications featuring a wide variety of LMWC are slowly gaining momentum, thanks to the increasing availability of diverse sample preparation methods, microcolumns and user‐friendly instrumentation. This work aims to present an illustrative guide for those interested in the implementation of LC/ESI‐MS bioanalytical methods tailored for LMWC using flow rates from 1 to 100 μL/min.

## 2 MINIATURIZED LC/ESI‐MS: THEORETICAL CONSIDERATIONS

The emergence of MS‐based metabolomics and proteomics in the early 2000s sparked renewed interest in low flow setups within the scientific community. In these fields of application, sample availability is often limited to a few μL, necessitating the adoption of microfluidic approaches throughout the entire analysis. This is because the reduction in column ID increases sample peak concentration, expressed as *C*_max_ = mN^½^/(2π^*½*^) ∙ *V*_0_(1 + k) where *m* is the absolute mass load of the analyte at retention factor *k* on a column with efficiency *N* and dead volume *V*_0_ (Abian et al., 1999). Therefore, a constant mass load with a reduction in column ID from 2.1 to 0.3 mm can result in an approximately 40‐fold increase in *C*_max_. To maintain identical chromatographic performances, the reduction of column diameter inherently leads to a decrease in injection volume and in the corresponding peak volumes of the separated analytes. These statements, however, hold true only if the analyte of interest is located at the column head after injection as a narrow band (e.g., without overloading, splitting, diffusion, and so forth; see Section 3.3 for considerations on injection volumes). The optimal detector volume, in this context, should not exceed one peak standard deviation *σ_V_*. This relationship is defined by the equation *σ*_*V*_ =* V*_col_ ∙ (1 + *k’*)/*N* ^*½*^, where *V*_*col*_ represents the column volume, *N* is the column efficiency in isocratic mode, and *k’* is the apparent retention factor (Desmet & Eeltink, 2013). As ion transport, mass analysis and detection in a mass spectrometer proceed on a different time scale (ms to μs) compared to chromatographic elution (s), this requirement is satisfied for LC/ESI‐MS (Spaggiari et al., 2013; Vanderlinden et al., 2016). However, mass spectrometers produce analytically useful (e.g., suitable for quantitation) signal based on mass flow, specifically the absolute number of ions hitting the detector. In theory, it should inevitably lead to a lower signal‐to‐noise ratio as the number of analyte ions is limited by a very small amount of injected material (Desmet & Eeltink, 2013; Poppe, 1997). From this perspective, chromatographic miniaturization alone may not necessarily yield the desired sensitivity increase, as the gain in analyte peak concentration is undermined by a decrease in flow. This behavior is typically observed at very low flow rates (generally <100 nL/min) (Abian et al., 1999; Bonvin et al., 2012; Marginean et al., 2008). In practice, a decrease in flow rate demonstrates an opposite effect for ESI/MS, dramatically increasing the available signal. This phenomenon is rooted in different efficiencies of ion production and sampling associated with various ionization source designs and chemical properties of the analytes.

### 2.1 Electrospray in low‐flow regime

An enhanced ESI response and following sensitivity gains depend both on the ion source design and the chemical nature of the analyte resulting in different ionization efficiencies. Emitters of low‐flow ion sources are usually placed closer to MS inlet, thus expanding the part of the captured ESI plume. In addition, the diameter of the emitting capillary is reduced to stabilize the Taylor cone at lower flow rates. Consequently, reduced emitter dimensions facilitate the production of smaller droplets with a higher surface‐to‐volume ratio, enhancing the desolvation process and increasing the charge available to analytes (Maxwell et al., 2010; Reschke & Timperman, 2011; Yuill et al., 2013). The combination of improved ion sampling and enhanced ion generation significantly impacts the performance of the ionization source: for conventional interfaces only 1 out of 2 × 10^5^ analyte ions are typically detected by the MS detector. In contrast, nano‐ESI improves this ratio to 1/400, resulting in a 500‐fold better signal (Abian et al., 1999; Plumb et al., 1999). Actually, it is even possible to achieve sampling efficiencies close to 100%, using nanoflow regime and modification of standard ion optics. However, this is still not the case for microflow and analytical interfaces (Schneider et al., 2021).

The observed sensitivity gains in low flow regime may greatly vary between different analytes. It is therefore essential to briefly understand physico‐chemical considerations at play. Differences in ESI efficiency between molecules are primarily dictated by polarity, which determine its position within the charged droplets in the spray (Enke, 1997). Less polar analytes have a stronger affinity to the highly charged droplet surface, while solvophilic species preferably reside in the droplet interior (Gomez & Tang, 1994). Consequently, the failure to migrate to the surface and participate to the excess charge will translate to a lack of ESI response. As an example, peptides with the most hydrophobic side chains will usually display higher ESI responses compared to those containing less hydrophobic motifs (Cech & Enke, 2000; Liang & Paul, 1993). Interested readers can consult literature on ion production mechanisms in ESI in detail elsewhere (Cech & Enke, 2001; Konermann et al., 2013).

In the particular case of bioanalytical applications, mixtures of analytes with different physico‐chemical properties are frequent, leading to an increased competition for the droplet periphery. Phospholipid species (and complex lipids in general), a typical interferent in biofluids or tissues, provide an excellent example. They usually display a high ionization efficiency and tend to outcompete other analytes at the droplet surface. It is thus expected that a low flow regimen will not dramatically enhance their ionization. In contrast, it may simply diminish their capacity to interfere at ionization with other coeluting molecules in the same polarity range, such as steroids or endocannabinoids. For instance, Danne‐Rasche et al. showed that employment of nanoflow in lipidomic analysis was able to enhance the ionization of low abundant co‐eluting isomers, increasing the total number of identified lipid species up to a factor 4 compared to conventional UHPLC methods (Danne‐Rasche et al., 2018). On the other hand, solvophilic analytes, such as glycosides, would also greatly benefit from low flow approaches. Nanoflow has indeed proven essential for the measurement of underivatized neutral oligosaccharides in ESI‐MS. Bahr and colleagues demonstrated a better ionization of maltopentose in nanoflow conditions, where competition with peptides for the droplet surface was diminished (Bahr et al., 1997; Karas et al., 2000).

### 2.2 Categorization of microflow setups

**Figure 2:**
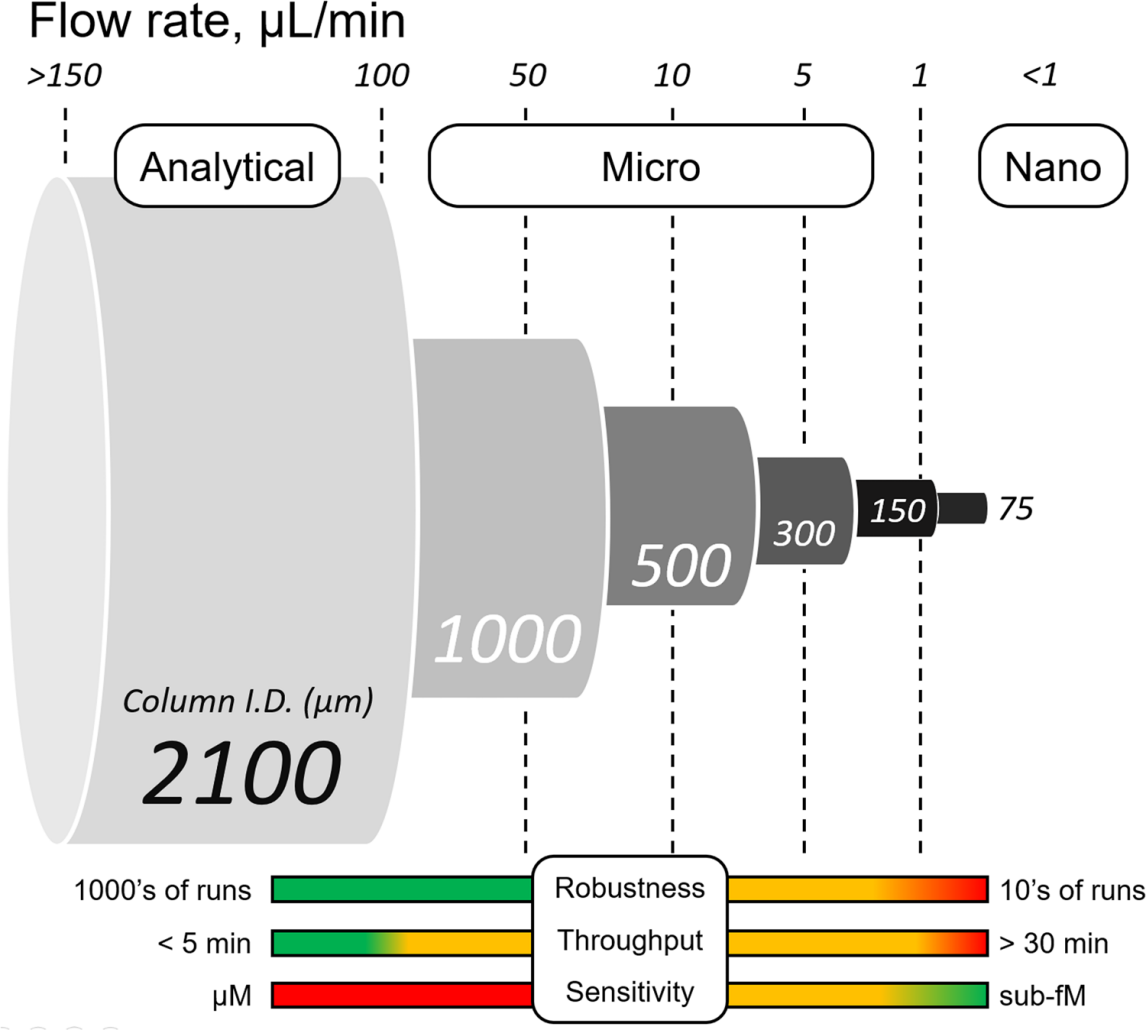
Fundamental assumptions regarding the robustness, throughput and sensitivity of liquid chromatography/electrospray ionization mass spectrometry setups considering the different flow rates range with column dimensions. [Color figure can be viewed at wileyonlinelibrary.com]

In general, the commonly used approach to categorize LC/ESI‐MS setups is solely based on column ID (Bian et al., 2022; Saito et al., 2004). Here, we prefer to emphasize flow rates, which share a strong relationship with the electrospray capillary ID to obtain maximum sample utilization efficiency (Bonvin et al., 2012). Flow rate serves as an immediate indicator for the expected signal improvement of the method under development compared to the established approaches (Figure 2).

From Figure 2, it becomes immediately clear that while the notions of robustness and throughput bear a certain degree of correlation and are proportional to the flow rate, they come at the expense of sensitivity. The primary impact on robustness at lower flow rates stems from reduced IDs of columns, liquid connections and emitter in the sample flow path, more prone to clogging and damage (Hilhorst et al., 2014). Throughput in turn depends on a careful management of extra‐column volumes in the system, such as injectors, valves and fluidic connections, which can significantly impact the run time (Desmet & Eeltink, 2013). We argue that microflow regimes may represent the best compromises in terms of overall performances by providing a strategic combination of high throughput and sensitivity with acceptable robustness. Moreover, any apparent lack of robustness can be offset by a scrutinized development of sample preparation protocols and by following the equipment operating procedures.

## 3 TECHNICAL DETAILS OF MINIATURIZED LC SYSTEMS FOR ESI‐MS

### 3.1 Mobile phase delivery

**Figure 3:**
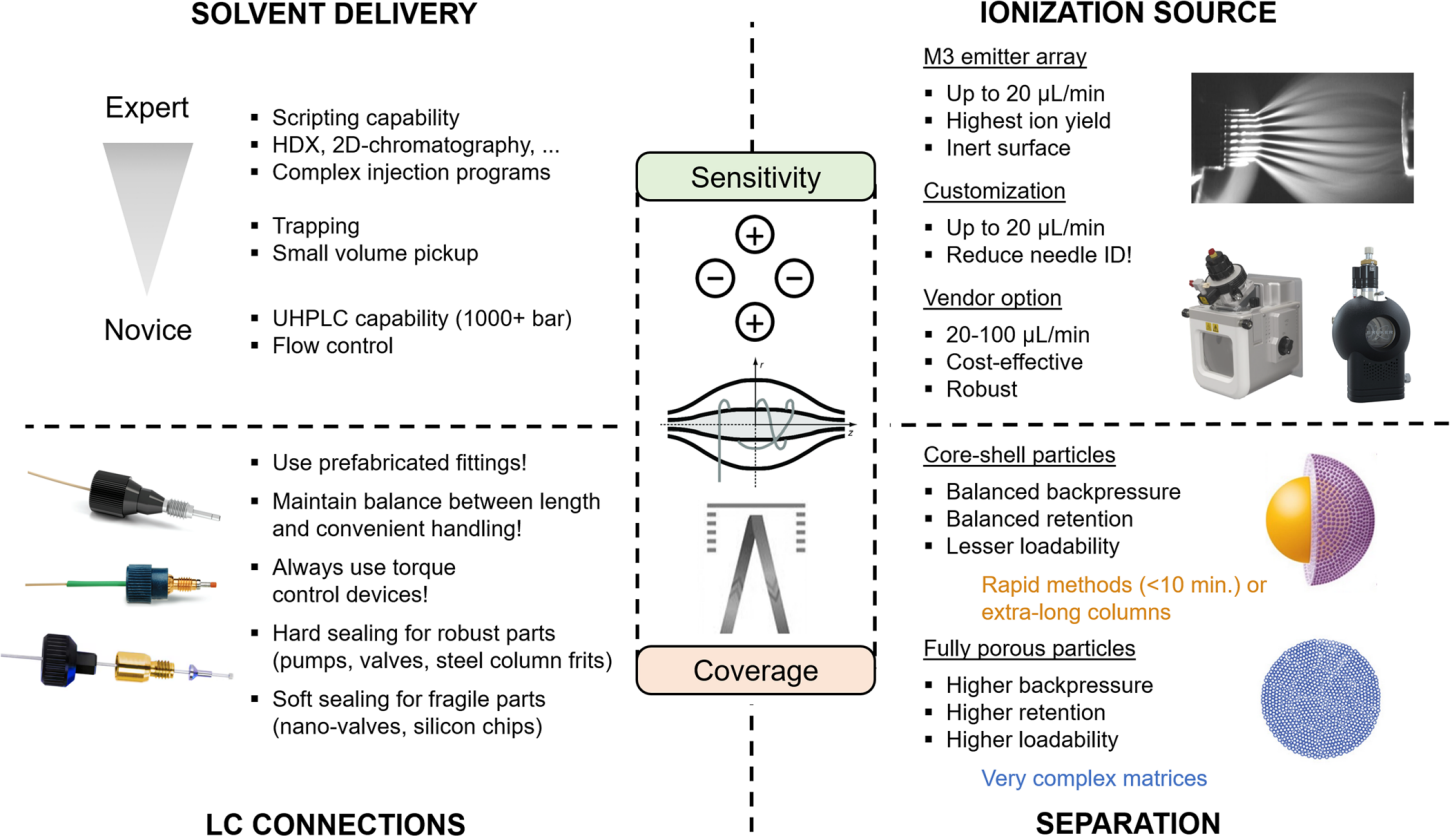
A graphical summary of the important hardware features to consider when planning microflow experiments. [Color figure can be viewed at wileyonlinelibrary.com]

Accurate, reproducible, stable and pulse‐free low flow is imperative for the functionality of microscale LC/ESI‐MS setup. Such flow can be generated using various devices, operating in either constant pressure or constant flow modes. Today, the predominant choice for commercially available user‐friendly systems involves piston‐based binary pumps coupled with a flow controller. In these setups, the flow controller constantly measures actual outputs at the outlet of the pumps plus the total flow rate and establishes a feedback loop for a precise piston motion control, ensuring a stable flow down to the nL/min range. In typical microflow working conditions, between 5 and 30 μL/min for column diameters of 0.3 and 0.5 mm, the resulting smallest flow to be delivered by one pump will vary from 0.25 to 1.5 μL/min, considering 5% of strong mobile phase at the run start. The combination of binary pumps with an active flow controller is thus crucial to deliver robust gradients (Figure 3). In addition, the delay volume of these pumps is extremely reduced to avoid excessive method runtime and loss of chromatographic resolution. Table 1 provides a concise overview of the technical specifications for some commercially available systems designed for microflow applications. Additional details, including the technical specifications of discontinued, but still widely used microflow chromatographs, as well as emerging technologies, can be found elsewhere (Nazario et al., 2015; Šesták et al., 2015).

**Table 1:** Technical specifications of currently marketed pumping systems for μLC/ESI‐MS from major vendors.

Vendor	Thermo Fisher	Waters	Sciex	Shimadzu	Bruker
Brand	Vanquish Neo	M‐Class	M5 MicroLC	Nexera Mikros	NanoElute 2
Design	Serial dual piston	Serial linear drive (2x)	Air pressure	Serial piston	Syringe
Gradient delivery	Binary				
Flow rate, μL/min	0.01–200	0.2–100	1–200	1–500	0.05–2
Active flow control	Yes	Yes	Yes	No	Yes
Pressure limit, bar	1500	1000	690	800	1000
Operating pH	2–10	2–10	1–10	2–10	2–10
Solvent degassing	Wash	Wash, A2/B2 channels	n/a	Separate module	n/a
Delay volume	<0.5 μL	<2 μL	<3 μL	<2 μL	<2 μL
Flow precision calibration	Yes	Yes	No	No	Yes

### 3.2 Fluidic connections

The impact of band broadening is critical when working at low flow rates and should be minimized to the greatest extent possible. Band broadening can be expressed in terms of variance as a sum of contributions from various parts of the setup such as *σ* ^2^_tot_ =* σ* ^2^_column_ +* σ* ^2^_injector_ +* σ* ^2^_tubing_ +* σ* ^2^_detector_ (Deridder et al., 2018; Desmet & Eeltink, 2013; Prüß et al., 2003). In case of gradient elution, the primary significant term is *σ* ^2^_tubing_, and more specifically the tubing located between the column and mass detector (including the electrospray capillary). It can be calculated using Taylor‐Aris equation as *σ* ^2^_tubing_ = *r* ^*4*^ *lπF/384* ∙ *D*_*m*_, where *r* and *l* represent the radius and length of capillary, *F* is the flow rate and *D*_*m*_ is the diffusion constant of the analyte in the mobile phase (Bello et al., 1994). Contributions from precolumn volume are effectively eliminated by gradient focusing effects (see Ch. 3.3), while mass spectrometric detection exerts no influence as ion transfer times from the inlet to detector proceed on a different timescale as outlined in Ch. 2.

Originally, establishing reliable fluidic connections posed a significant challenge in adopting microflow, requiring skilled operators to manually produce polished fused silica capillaries of required dimensions. Nowadays, various suppliers offer prefabricated fittings, ensuring leak‐free connections and effectively eliminating connectivity issues. Table 2 provides a summary of currently available fittings to connect the components of microflow setups in the established 1/16″ (10/32 thread) format. To prolong the life of equipment and ensure reproducibility of the fluidic connections, it is strongly advised to use torque control while creating the connection. In our experience, 0.35–0.4 Nm torque wrench works well with nanoVipers and Waters ZenFit connectors, requiring only the head piece to be produced independently by creating a slit for the capillary in a bar with a column head geometry. We however advise against using Marvel XAct fittings with embedded torque control on fragile parts such as silicon chips or nano‐valves. Of note, a rather strong effort is required with hard PEEK sealings to produce a haptic response and leak‐free connection (Figure 3). Finally, it is worth keeping attention to which materials are used for needles, flow path, valves and other parts coming in contact with the analytes to avoid nonspecific adsorption. In most cases, MP35N and PEEKsil will be sufficient. The latter is also used to build prefabricated fittings of suitable IDs, which should be used to connect all the parts of the setup.

**Table 2:** Prefabricated fittings for user‐friendly fluidic connections of μLC/ESI‐MS setups from major vendors.

Vendor	Thermo Scientific	Waters	IDEX
Brand	nanoViper	ZenFit	Marvel XACT^a^
Sealing	Soft PEEK^b^	Vespel	PEEK
Material, µm ID	PEEKSil^c^ 20, 50, 75 100 MP35N 100	PEEKSil 25, 40, 75, 100	PEEKSil 25, 50, 75, 100 PEEK‐lined stainless steel 25, 50, 75, 100
Length, mm	150, 250, 350, 500, 750, 1000	250, 500, 650, 750, 1500	100, 150, 300, 500, 750, 1000
Backpressure, bar	1200, 1500	1000	1300
Fingertight	Yes	Yes	Yes
Torque limiting	Wrench^d^	Wrench^d^	Yes
pH range	1–10	1–10	1–10

a Same fittings are offered by Phenomenex as SecurityLink™ and Bruker for fluidic connections of NanoElute LC devices.

b Polyether etherketone.

c PEEK‐lined fused silica.

d Available from Thermo,3rd party LC/MS equipment vendors or to be produced in‐house.

In practice, it is essential to keep the ID and length of the tubing as small as possible for the available backpressure. All connections should be made using zero dead volume (ZDV) connectors. Typically, 25 μm ID capillaries are suitable for nanoflow and microflow setups operating up to 5 μL/min, 50 μm ID capillaries for the 5–50 μL/min methods and 75 μm ID for the higher flow rates. We generally recommend using tubing lengths of 25–50 cm to achieve an optimal balance between minimizing dead volumes and ensuring ease of handling. As an example, our Waters M‐Class LC setup typically operating at 2–5 μL/min comprises a 50 cm 25 μm Marvel XAct fittings for pump‐to‐autosampler and valve manager‐to‐column sections, and a 35 cm piece of the same dimensions for autosampler‐to‐valve manager connection. Column and M3 emitter are, in turn, linked via 25 or 35 cm 20 μm nanoViper capillary, as per manufacturer specifications. For the presented system, the pressure contribution from the fluidic connections typically ranges between 100 and 300 bars, depending on solvents and flow rates.

### 3.3 Sample introduction

Lower flow rates necessitate the reduction of column dimensions. Consequently, smaller injection volumes are needed to strictly maintain chromatographic performance (Desmet & Eeltink, 2013; Vissers et al., 1997). For instance, a 300 μm ID column accommodates 0.04 μL of sample, equivalent to a 2 μL injection in an analytical setup (2.1 mm ID). However, such volume is already not available on most of the commercial instruments (typically min. 0.1 μL injection). In the context of nanoflow arrangements, equivalent injection volumes become even less practical. Although it is technically possible to provide on‐column injections in single nanoliter volumes using specialized valves and/or custom‐made injection loops, this technique is seldom used (Lubin et al., 2017; Vissers et al., 1996).

Employing relatively large injection volumes becomes possible as miniaturized bioanalytical applications predominantly use gradient elution mode. When the analyte is injected in a solvent corresponding to a weaker composition than that needed for its isocratic elution, it's allowed to be focus as a thin band at the column head. Next, the elution occurs when gradient composition provides sufficient solvent strength (Ling et al., 1992). The injection volume for a microflow setup should not exceed 40% of the effective column volume (*V*_col_) to avoid losses in chromatographic efficiency. However, minimal impact of the injection volume on strongly retained compounds on a C_18_‐based 0.3 mm ID column was observed when the injection band volume reached 160% of *V*_col_. By contrast and as anticipated, a significant reduction in peak capacity occured primarily for weakly retained substances or those eluting in the void volume (Werres et al., 2021).

Commercial instruments commonly employ fixed split‐loop injectors, which aspirate an exactly specified aliquot of the sample bracketed by an injection solvent through a direct connection of a needle, sample loop and metering device (De Vos et al., 2016). These injectors can operate in either partial or full loop mode. The latter employs an overfill technique, ensuring maximal reproducibility, but at the cost of a higher sample consumption, typically 3–6 times the desired injection volume. In contrast, the partial loop mode sacrifices some accuracy and precision in favor of increased flexibility in method development. For microflow applications at 20–100 μL/min range, where an impact on gradient delay is less pronounced, flow‐through‐needle (FTN) injectors may be employed at a cost of increased band broadening effects (Broeckhoven et al., 2018; Deridder et al., 2018).

An alternative method of sample loading is related to the column switching approach. Here, a higher volume of the sample is introduced at higher flow rates (up to several hundred μL/min) onto a trapping column typically packed with large particles featuring high affinity for the analyte using a separate loading pump. Subsequently, matrix components, salts and impurities are either washed out with a loading solvent or selectively removed from the trap, if chromatographic separation on the trapping column is technically feasible. Next, analytes are transferred to the analytical column using a low‐volume switching valve, either in front flush (analyte travels through the trap) or back flush (analyte eluted from the trap head) mode. To further increase the reliability of the system, a filter can be installed between the injector and the trap to capture particulate matter. The captured particulates are then flushed to waste in the backward direction using an additional pump and a system of switching valves. For microflow configurations, it is advisable to avoid transferring a single analyte plug from the trapping column with a strong solvent, as the volume of such a plug may be significant in comparison to the volume of the analytical column, potentially causing breakthrough. In addition, the retentivity of the trapping stationary phase should be lower compared to that of the analytical column to prevent excessive band broadening (Coppieters et al., 2021; Rochat et al., 2021; Rogeberg et al., 2014). The trap‐and‐elute configuration thus enables to make large volume injections (in the range of 10–20 μL on 0.3‐ and 0.15‐mm ID columns) while minimizing problems related to particulates and loading capacity. It however requires the purchase of additional switching valves and auxiliary pumps.

### 3.4 Chromatographic columns

Chromatographic columns for low flow setups can be categorized into two main groups: packed columns, filled with a suitable chromatographic material, and open tubular columns. In the first group, which is the most prevalent type in contemporary LC devices, columns are typically made of stainless steel or supported fused silica hardware. These columns are equipped with frits and filled with particles carrying a dedicated chromatographic functionality (C18, phenyl, amino, etc.) (Perchepied et al., 2022). This design offers high backpressure resistance (up to 1500 bars), substantial sample loading capacity and the flexibility of employing various modes of interaction to separate different analyte groups (Kuehnbaum & Britz‐McKibbin, 2013). The second group, encompassing open tubular columns, holds significant promise in chromatography. These columns achieve separation either in the porous layer of the column wall or on the wall coating layer (Mejía‐Carmona et al., 2020). They exhibit characteristics such as high permeability and extraordinary chromatographic efficiencies in the order of multiple 10^5^ of plates per meter. Contrary to gas chromatography, where such columns are regularly employed, only a few applications of those currently exists for LC, being primarily driven by fundamental research. These columns typically operate at extremely low flow rates due to their very narrow ID (1–5 μm) and specific sample loading conditions. Importantly, they require highly customized instrumentation for their effective use. Given the special nature of these columns, we will not cover this topic here, but instead encourage the readers to the relevant literature for further exploration (Lam et al., 2019; Mejía‐Carmona et al., 2020).

Another design which shows promise for microscale applications are monolithic columns. Such columns are prepared in situ by means of polymerization of a mixture of a suitable monomer and a porogen solvent. Size and morphology of the pores depend on properties of both counterparts and polymerization kinetics, whereas selectivity is provided by the type of monomer and functionalization (Premstaller et al., 2001; Urban & Jandera, 2008). The result is typically a continuous sponge‐like bed, featuring macro‐ (1–10 μm), meso‐ (5–20 nm), and micropores (<5 nm). Macropores facilitate eluent flow‐through, whereas mesopores provide a surface with active adsorption sites to facilitate the separation process (Moravcová et al., 2003). Although these columns provide better efficiency for macromolecules due to their porosity structure, it is possible to obtain higher efficiencies also for LMWC. A special interest of these columns for miniaturization comes from the ease of preparation in capillary format, absence of frits and higher permeability. Combined together, these properties allow for a robust long column with exceptional kinetic performance (Ishizuka et al., 2000; Minakuchi et al., 1996). Despite these advantages, monolithic columns, after having gained momentum in the early 2000s, have become less widespread due to their lack of batch‐to‐batch reproducibility and are mainly available today via in‐house synthesis. The only remaining major commercial provider for capillary and μLC formats is GLSciences (Japan). Interested readers can refer to the following literature for an in‐depth view on the subject (Svec, 2017; Svec & Lv, 2015; Unger et al., 2008).

Chip‐based columns constitute another prominent family of separation devices for μLC and can be considered by extension as silica monoliths. These columns are produced from silica using a photolithographic process similarly to the production of microprocessors, to create a channel for subsequent packing (Thurmann et al., 2015; Yin & Killeen, 2007) or an array of ordered pillars. The pillars are then etched and modified with a suitable ligand to form a stationary phase (He & Regnier, 1998; Vankeerberghen et al., 2023). Such pillar array columns, initially developed by the group of Desmet (Detobel et al., 2010) has been commercialized under the brand of μPAC^TM^ by Thermo Scientific. The advantages of these columns are lower flow resistance, excellent reproducibility of retention times and high efficiencies. The first generation of μPAC^TM^ featured 5 μm pillars separated by a 2.5 μm distance, whereas these values are halved on the second generation (Vankeerberghen et al., 2023). Despite their merits, their widespread adoption in bioanalysis is constrained by their cost and limited choice of stationary phase chemistries and dimensions. Nevertheless, successful applications of μPAC^TM^ in proteomics and lipidomics have been reported, with anticipation of more applications in the future (Rozing, 2021).

In the present review, we focus on packed microcolumns, due to their well‐established technology and consistent market availability. It is generally admitted that these columns prove ideal for long‐term routine method development, owing to their user‐friendly nature, robust technology, ample sample loading capacity, and reliable lot‐to‐lot reproducibility. While μLC columns may exhibit lower chromatographic efficiency than conventional columns, it is worth highlighting that the primary goal of low flow methods is signal amplification rather than outstanding chromatographic performance. Besides, compared to their analytical counterparts, capillary columns suffer less from frictional heating and wall effects, which might partially compensate for the lower chromatographic efficiency (Gritti & Wahab, 2018; Gritti et al., 2016). Although columns with smaller IDs are more limited in terms of particles chemistries, the μLC niche has not been completely overlooked by the market, and there is a good offer of relevant column formats bonded with various ligands. Table 3 summarizes relevant products offered by major vendors in a timely and reproducible fashion. The emphasis is on modern, highly efficient packings with fully (FPP) or superficially (SPP) porous particles, featuring standard connections designed to withstand higher backpressures (Figure 3). Besides, the advantage of lower flow resistance of superficially porous particles can be easily exploited in µLC setups, as higher linear velocities are conveniently accessible with μLC pumps. This approach proves particularly fruitful for high throughput methods, minimizing losses in resolution while enabling shorter analysis time (González‐Ruiz et al., 2015; Tanaka & McCalley, 2016). As an illustration, the transfer from a 2.1 × 150 mm column (2.7 μm) at 1 mL/min to a 0.5 × 150 mm column would allow for approximately 60 μL/min flow rates at 850 bars while maintaining most of the method resolution and greatly increasing detection limits.

**Table 3:** An overview of well‐established columns with high kinetic performance (sub 2 μm or sub 3 core shell) for microscale LC/ESI‐MS analysis (major vendors, as of 01.11.2023).

Vendor	Waters	ChromaNik	AMT	Phenomenex	YMC	Thermo Scientific	Bruker
Brand(s)	nanoEase MZ Acquity UPLC	SunShell	Halo	Kinetex Luna Omega	‐	Hypersil Gold uPac Neo	PepSep
Length, mm	50, 100, 150	50, 150	50, 100, 150	50, 100, 150	50, 100, 150	55, 50, 100	50, 80, 100, 150, 250
Hardware	Stainless steel	Steel, PEEK/fused silica	Steel, PEEK/fused silica	Stainless steel	Steel/fused silica	Steel, Silicon (μPAC)	PEEKSil
Column ID, μm	Available column chemistry						
150	‐	‐	‐	‐	‐	μPAC C_18_	C_18_, C_18_‐Aq
200	‐	C_18_, Phenyl	‐	‐	‐	‐	‐
300	BEH, CSH, HSS T3^a^		All major chemistries^b^	All major chemistries	All Triart phases		
500	‐						
1000	All major chemistries			Biphenyl, C_18_, Polar C_18_	C_18_	C_18_, C_18_‐Aq, C_8_	
Particle size, μm	1.7, 1.8 FPP	2.0 SPP	2.7 SPP	1.6 FPP, 2.6 SPP	1.9 um FPP	2.5 pillar, 1.9 FPP	1.5, 1.9 FPP
Backpressure, bar	1000	800	600	1000	600	1000	1000
Temperature, °C	80	80	50	50	80	50	60
Guard	filter 0.2 μm, steel	n/a	n/a	Filter 2 μm, steel	Yes	Yes	n/a
Trap	For 300 μm C_18_	n/a	n/a	For C_18_ phases	Guard	Yes	Yes

a All phases feature C18 ligand: BEH, bridged ethylene hybrid; CSH, charged surface hybrid; HSS T3, high‐strength silica with low‐density triple‐bound ligand.

b Recently introduced stationary phases may be unavailable.

The majority of the presented columns prominently feature various types of C_18_ ligands, primarily tailored for RP separations. This choice aligns with the versatility of the RP approach and its historical application in the separation of tryptic digests in bottom‐up proteomics (Lenčo et al., 2022). Nevertheless, alternative separation modes such as hydrophilic interaction liquid chromatography (HILIC) are also available in miniaturized formats. Several vendors, particularly Merck (ZIC), AMT and YMC (Diol), offer columns tailored for HILIC applications. The utility of HILIC phases is somewhat limited due to the absence of readily available hardware with reduced nonspecific adsorption. Hence the analysis of polar compounds with increased sensitivity requirements, such as certain pesticides, phosphorylated peptides/lipids and oligonucleotides, is hindered by their capacity to bind to metal surfaces (Guimaraes & Bartlett, 2023). A monolithic capillary column with embedded amide functionality in PEEK hardware (GL Sciences; MonoCap Amide) may represent an interesting commercial option in this particular case.

Modern μLC columns exhibit robustness enabling them to withstand frequent pressure fluctuations of varying frequency and amplitude, as outlined in their specifications. However, it is strongly recommended to avoid rapid pressure drops and unnecessary decompression, considering the overall higher fragility of miniaturized instrumentation. The absolute loading capacity of microcolumns is significantly lower compared to conventional ones, making matrix build‐up effects more pronounced. To ensure a reliable method able to handle large batches, a practical rule of thumb is “to elute everything towards the end of the gradient.” Given the constraints of miniaturized systems, rapid switching between mobile phases is unfeasible. Therefore, it is advised to employ stronger eluents in combination with more gentle gradient slopes to provide column wash of sufficient strength.

### 3.5 Ionization sources and mass spectrometry considerations

The ionization source plays a pivotal role in any microflow setup, defining the efficiency of ESI and, consequently, the sensitivity improvement. Furthermore, the spray characteristics can be significantly influenced by the material and geometry of the emitter (Maxwell et al., 2010; Reschke & Timperman, 2011). The fundamental concept behind the design of any microflow ionization source is to minimize the extra‐column volume and the dimensionality of the sprayer. In the majority of cases, sensitivity improvements compared to the classical approach may be achieved using flow rates between 50 and 100 μL/min on core‐shell 0.5 mm or fully porous 1.0 mm ID columns. In this case, the MS vendor's microspray option is the most practical and economical choice (Figure 3). Typically, such options are provided by the MS instrument vendor as a separate device (e.g., Shimadzu, Sciex) or as the possibility to replace the conventional ESI needle with one of a smaller ID (usually 50 µm needle) in the case of Bruker (Midha et al., 2023), Thermo Scientific and Waters. For lower flow rates, a customization of the ionization source is required by forging the needle insert with the reduced ID, for example, EASY‐Spray Jailbreak (Phoenix S&T; Thermo), microflow ion source (Prolab GmbH, Switzerland; Sciex) and DuoESI (Newomics; Thermo, Bruker, Agilent, Shimadzu). The latter is of a particular interest, featuring a unique microfabricated monolithic multinozzle (M3) sprayer, that divides the incoming flow into multiple streams, transitioning from microflow LC to nanospray (Kim et al., 2007; Mao et al., 2011). This design has a potential to maximize sensitivity by combining higher mass loads with the most efficient ionization. An alternative involves an integrated approach, combining fluidic connections, a heater, a column and a sprayer into a single module (Broccardo et al., 2013). Represented by devices such as ionKey (300 and 150 μm ID; Waters) and EASY‐Spray (150 μm ID; Thermo), this family offers a key advantage with practically nonexistent extra‐column volume. The embedded column is directly connected to a sprayer, resembling nano‐LC setups, and produces sharply defined chromatographic peaks. Samples are typically loaded onto such integrated devices via trapping, providing additional protection for this otherwise expensive part.

In all scenarios, some degree of manual optimization is inevitable. The primary objective is to achieve a stable spray as close as possible to the sprayer inlet, ensuring maximum sampling of the stable ESI plume into the spectrometer (Bonvin et al., 2012). Simultaneously, efforts are directed towards minimizing adverse effects such as analyte deposition resulting from sprayer overheating, electric discharges, and fouling (or even blockage) of the ion transfer region with residues of complex matrices, as demonstrated by Rochat et al. (2021). These detrimental effects have to be reduced to the absolute minimum to guarantee a reproducible analysis of large batches.

Finally, the increased ion currents resulting from the combination of μLC with the optimized ionization source will lead to increased wear of ion optics and detectors within the mass spectrometer. Logically, a more frequent cleaning of ion optics may be necessary. In contrast with triple quadrupole (QqQ) and orbitrap mass spectrometers which feature spatial separation and management of the incoming ion current by design, multichannel plate detectors of time‐of‐flight (TOF) instruments may be prone to a quick saturation (Liu et al., 2014; Westman et al., 1997) and rapid detector wear if ion current control measures are not enabled or implemented (Ibrahim et al., 2008; Syka et al., 2004). On the other hand, TOF instruments feature better analytical coverage compared to trap‐based mass analyzers due to the absence of space charge capacity limitations.

## 4 SETTING UP A ROBUST ΜLC/ESI‐MS WORKFLOW

### 4.1 Methodological considerations

**Figure 4:**
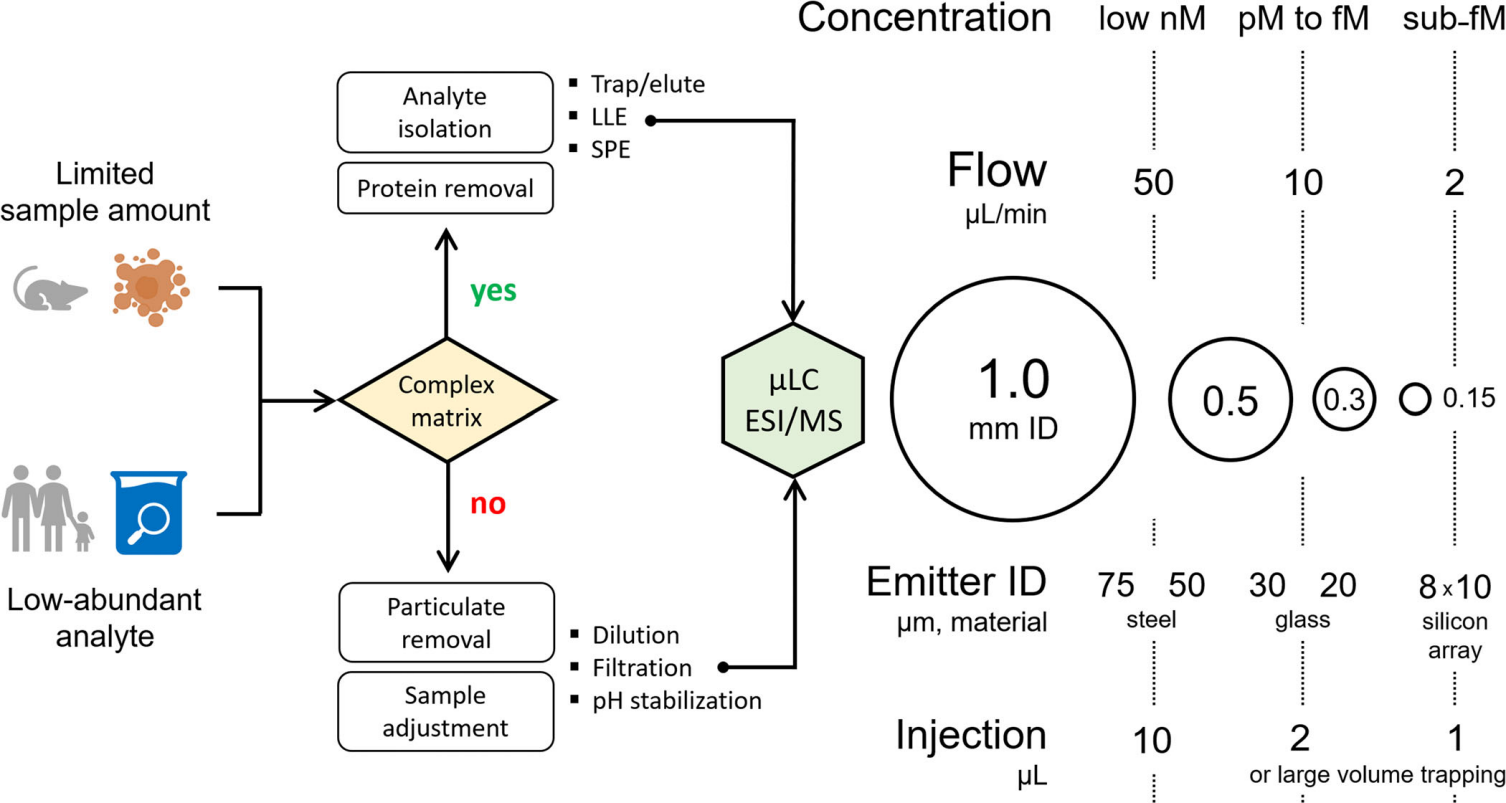
An approach to a rational selection of miniaturized liquid chromatography/electrospray ionization mass spectrometry setup based on targeted analyte concentration. [Color figure can be viewed at wileyonlinelibrary.com]

As mentioned in Ch. 3.4, the major driver for employing miniaturized LC/ESI‐MS coupling stems from the necessity to reliably detect low‐abundant analytes within a relatively large sample matrix. Therefore, the evaluation of the applicability of μLC/ESI‐MS, beyond equipment availability, hinges on the convergence of the chemical properties of the matrix/analyte combination and the desired detection levels of the method. When dealing with very low‐abundant analytes, a systematic approach should be adopted in designing a prospective microflow setup. To assist in this decision‐making process, we provide a scheme that succinctly outlines methodological considerations for microflow setups, considering the desired sensitivity levels and compatible sample preparation techniques to handle matrix complexity. Our ultimate aim is to achieve the highest possible sensitivity while maintaining chromatographic performance. Therefore, it is essential to account for the total mass load when analyzing biological matrix. Matrix interferences will inevitably compete for the available stationary phase adsorption sites thus reducing the loading of the target analyte compared to injections in a neat solution. Consequently, biological samples may require appropriate dilution or adjustment of the preconcentration factor during sample preparation (Figure 4).

Importantly, the targeted or nontargeted nature of the application should be considered. Targeted and semi‐targeted analysis typically focus on a limited number of analytes, involving specific sample preparation procedure with extensive cleanup. Conversely, nontargeted studies aim to encompass a broad sample chemical space, leading to increased contaminants and matrix compounds due to simplified sample preparation methods, such as protein precipitation (plasma) or simple dilution (urine). For nontargeted analyses requiring minimal sample preparation in complex matrixes, it is preferable to use 20–80 μL/min flow rates with columns of 0.5‐ or 1.0‐mm ID. This analytical approach is particularly effective when paired with stainless‐steel emitters of relatively large ID (50–75 μm). This combination minimizes the impact of matrix components during continuous analysis of large batches, thus increasing system robustness. Although it is possible to inject samples prepared in the nontargeted fashion onto columns of smaller ID at lower flow rates to further increase the analytical signal, special precautions should be taken during sample preparation (e.g., suitable solvent composition to avoid precipitation, avoiding overload, compulsory filtration before injection, etc.).

We cannot emphasize enough that maximal sample cleanup should be a continual objective in low and capillary µLC/ESI‐MS measurements. First, it is imperative to eliminate non‐volatile compounds such as salts, as they can undesirably accumulate on the emitter tip. Second, considering the limited loading capacity of microcolumns, the ideal sample should exclusively consist of the analyte to fully occupy the available particle surface. For complex biological matrices, such as plasma, serum or seminal fluid, desalting, deproteinization and delipidation are crucial. This can be achieved through salt‐assisted liquid–liquid extraction (SALLE) (Liu et al., 2019), or a carefully designed elution from SPE cartridge (David et al., 2014; Seidi et al., 2019). To eliminate protein residues, a protein crush step before SPE is essential (Thomas et al., 2013). We recommend to allow for overnight precipitation at a temperature of at least −20°C and repeat the centrifugation step twice before injection. Alternatively, LLE is a fast and easy to handle method that typically eliminates salt, protein or lipid transfer into the sample (Shen et al., 2013; Souverain et al., 2004; Zardini Buzatto et al., 2020), although it is less suitable for polar compounds. Miniaturization works well for LLE, allowing for a highly favourable solvent‐to‐sample ratio. For an optimal cleanup, mixed‐mode SPE may provide the best solution due to the variety of interactions involved (Gilar et al., 2008; Nadal et al., 2022).

Following the reconstitution step, it is recommended to filter the sample using a spin filter or a well plate of at least 0.2 μm to prevent particulates in the narrow channels of the injector and autosampler. Molecular weight cutoff filters present an even better alternative, enabling selective deproteinization. However, filtration times may be prolonged, especially for 3K‐cutoff filters and/or in presence of high viscosity solvents or when filtering at lower temperatures. Finally, an attention should be given to potential nonspecific adsorption of analytes to the filter material and other surfaces.

### 4.2 Advantages and pitfalls of μLC/ESI‐MS: Theory becomes practice

**Figure 5:**
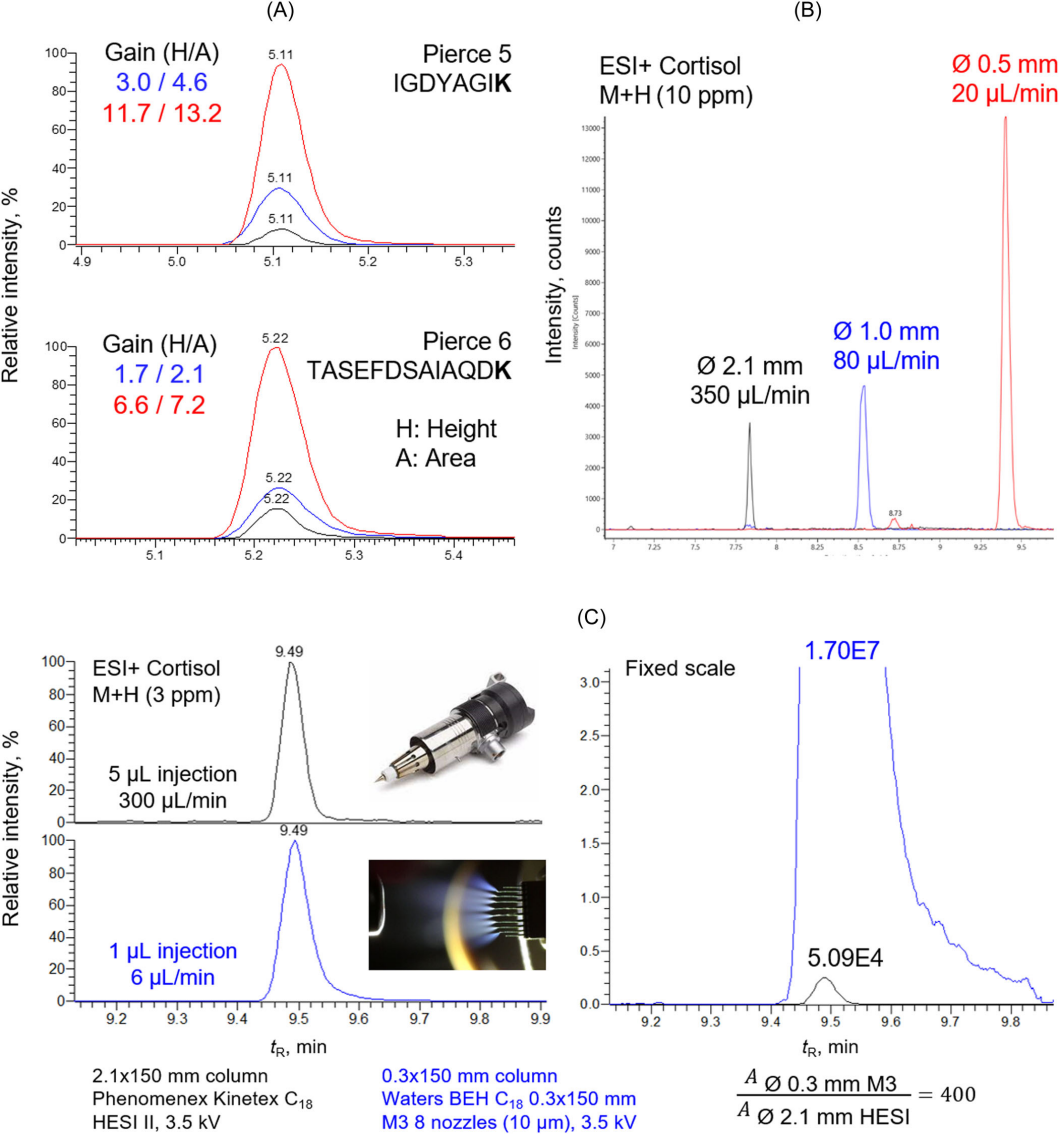
Selected ion chromatograms (SIC) of (A) Pierce® peptides, (B) endogenous steroid cortisol on 150 mm YMC Triart C_18_ columns with the corresponding reduction of flow rates, and (C) on a 0.3 mm ID column with novel M3 emitter versus analytical column with conventional HESI II source. [Color figure can be viewed at wileyonlinelibrary.com]

Based on our experience, a substantial 4–10× improvement in peak area can be consistently achieved with minimal challenges when transitioning from standard ionization sources to reduced ESI needles adapted to flow rates <100 μL/min (column ID 0.5–1.0 mm). Such benefit is illustrated in Figure 5A, where we analyzed the Pierce peptide mix using columns of identical length (150 mm) and chemistry, but differing in diameter to assess the performance of μLC/ESI‐MS. The improvement in sensitivity is evident, albeit with a slight trade‐off in chromatographic performance. For instance, the baseline width of the Pierce 5 peptide peak was 0.10 min on a 2.1 ID column, while it increased to 0.14 min for the 1.0‐ and 0.5‐mm ID counterparts. Interestingly, for the Pierce 6 peptide, while the first reduction of ID (2.1 > 1.0 mm) only provides a 1.7‐fold increase in peak intensity, the second reduction (1.0 > 0.5 mm ID) provides an over threefold increase. This can be unequivocally attributed to the use of a more suitable ESI emitter for the given flow rate (50 vs. 100 μm needle). Different physicochemical properties of both compounds may also contribute to intrinsic ionization efficiency, which explains uneven signal gains. In addition, the enhanced availability of charges may induce shifts between charge states in the microflow regime, particularly for analytes like peptides or natural products with multiple ionisation sites. Quantitative work should consider the charge state distribution, as well as the possible presence of newly formed adducts and their ratios. Similarly to small peptides, an eightfold improvement in peak area was obtained for LMWC in matrix conditions, as illustrated with cortisol in the course of nontargeted profiling of human plasma samples (Figure 5B).

From a practical standpoint, we realized that the ion source design plays a crucial role in the absolute improvement of the analytical signal and that precise optimization of ion sampling with original ion sources was challenging. *Y*‐axis adjustment was absent on the Waters instrument and HESI II on a Thermo QExactive machine faced arcing issues. Additionally, neither of the sources featured a camera and internal lamp to visualize the spray plume during analysis, hindering reasonable voltage/gas flow adjustments and emitter tip observation during analysis. Based on this experience, we took a significant step forward by employing a 0.3 mm ID column at a flow rate of 6 μL/min, with a novel M3 emitter on Orbitrap Exploris 120. This approach allowed us to simultaneously achieve increased ion yields, observe the spray and reduce nonspecific adsorption in the system due to the more inert material of the nozzles. Analyzing a depleted plasma sample prepared through multiple filtration and SPE after careful optimization revealed a remarkable improvement of more than two orders of magnitude for cortisol. However, an approx. 6x reduction in chromatographic peak capacity was observed due to the massive volumetric overload (1 μL on a 0.3 mm ID column corresponds to 50 μL on a 2.1 mm ID column) (Figure 5C). This last example shows all the potential of μLC/ESI‐MS compared to standard analytical conditions.

## 5 CURRENT BIOANALYTICAL APPLICATIONS AND METHODS FOR LMWC

In this section we present a compilation of studies spanning from 2018 to 2023 that have employed microflow approaches coupled to mass spectrometry for bioanalytical applications (Table 4). Despite the relatively small number of microflow applications focused on LMWC, several emerging trends can already be identified.

**Table 4:** Selected studies from 2018 to 2023 applying μLC/ESI‐MS approach in capillary to semi‐microflow regimes.

Workflow	Matrix	*V*_*biofluid*_, μL	*V*_*sample*_, μL	LC model	Column	Flow, μL/min	Detection limits, pM	MS model	Ref.
*Targeted quantitative analysis*									
Endocannabinoids	Cerebrospinal fluid	250	3	Waters nanoAcquity	Phenomenex C_18_ 150 × 0.3 mm (2.6 μm)	4	0.7–1293	Shimadzu 8060 (micro‐ESI) MRM, 30 μm ID TaperTip	He et al. (2022)
Peptide hormones	Human plasma	50	7 on trap	Waters M‐Class	Waters ionKey Peptide BEH 150 × 0.15 mm (1.7 μm)	3	4–200	Waters G2‐XS Q‐TOF, full scan Waters TQ‐XS, MRM	Chen et al. (2019)
Cortisol, cortisone	Saliva	700	5	Sciex Eksigent MicroLC 200	YMC Triart C_18_ 50 × 0.5 mm (3 μm)	20	28–56	Sciex QTRAP 4500 MRM, 50 μm electrode	Aydin et al. (2021)
Drugs & metabolites	Primate plasma	25	2	Sciex M5 MicroLC	AMT Halo C_18_ 50 × 0.2 mm (2.7 μm)	5.4	150–300	Sciex API6500 MRM, OptiFlow source	Barricklow et al. (2020)
Estrogen metabolites	Human serum	200	3 on trap	Waters nanoAcquity	Home‐packed BEH C_18_ 220 × 0.2 mm (1.7 μm)	1.7	60–1439	Sciex QTRAP 4000 MRM, 20 μm emitter	Kafeenah et al. (2022)
Drugs & metabolites	Neonatal plasma	25	0.5	Sciex Eksigent 2D Plus	Waters 100 × 0.15 mm (1.7 μm)	1	35–175	Sciex TripleTOF 5600+SWATH, PicoView 450 source	Xiao et al. (2021)
Peptide hormones	Diluted plasma	900	250 on trap	Thermo Scientific Ultimate 3000 RSLCnano	Diverse RP‐type 50–150 mm, 0.2–0.3 mm ID	10	2.5–250	Thermo QExactive Plus Full scan + DDA, M3 source	Rochat et al. (2021)
*Nontargeted and structural applications*							Improvements		
Profiling of urea cycle disorder mouse model	Mouse urine, brain	20 urine 20 brain	0.8	Waters M‐Class	Waters HSS SB C_18_ Imtakt Scherzo SM‐C_18_	86	Not specified	Thermo QExactive Full scan, 50 μm electrode	Geller et al. (2022)
Investigation of miniaturization impact on wide‐scale analysis	Human plasma	100	3	Thermo Scientific Vanquish Duo	Waters HSS T3 C_18_ 150 × 1.0 mm (1.7 μm)	57	Not specified	Thermo QExactive HF Full scan, 50 μm electrode	Fitz et al. (2022)
Identification of drug metabolites	Mouse plasma	75	5	Waters M‐Class	Waters ionKey BEH C_18_ 50 × 0.15 mm (1.7 μm)	3	Not specified	Waters G2‐XS Q‐TOF	Majewsky et al. (2018)
Investigation of HILIC in μLC conditions	Porcine kidney	tissue	1	Prolab Zirconium Ultra	Custom BEH amide HILIC 150 × 0.3 mm (1.7 μm)	5	80× more signal for bile acids	Agilent 6550 iFunnel QTOF Prolab micro‐ESI interface	Neef et al. (2023)
*Technological advancements*									
Coupling with differential ion mobility	Glycopeptides	2 μM solution	2	Shimadzu Nexera Mikros	AMT Halo C_18_ 100 × 0.5 mm (1.7 μm)	20	Not specified	Sciex TripleTOF 6600 + Micro probe, DMS cell	Jacquet and Hopfgartner (2022)
Coupling with vibrating sharp‐edge spray ionization	Tryptic digest Metabolites Nucleotides	Diverse	n/a	Flow splitting	Analytical LC to 0.36 mm ID fused silica capillary	30	Better ionization efficiency	Thermo QExactive Full scan, custom ion source	Majuta et al. (2021)
Coupling with atmospheric pressure chemical ionization	Acridine	50 μM solution	0.1	Sciex ExionLC AD	Agilent Zorbax XDB‐C_18_ 150 × 0.5 mm (3.5 μm)	10	10× more ion yield	Thermo LCQ Fleet ion trap Full scan, custom ion source	Strmeň et al. (2018)
Implementation of μ‐(2D‐LC) separation	Human plasma	200	0.5	Waters I‐class 2D configuration	100 × 1.0 mm BEH amide (1D) 150 × 1.0 mm HSS T3 C_18_ (2D)	100	Higher annotation rates	Waters Synapt G2‐Si Thermo LTQ Orbitrap XL	Orlandi et al. (2023)

First of all, it is obvious that these bioanalytical applications primarily target specific families of compounds and often utilize sample and injection volumes similar to those in established analytical setups. For instance, Aydin et al. achieved the analysis of cortisone and cortisol from saliva by subjecting 700 μL of this biofluid to an SPE on Oasis PRIME 96‐well plate, a widely used material in steroid analysis. Here, a relatively large injection volume of 5 μL was made onto a 0.5 mm ID column. Such volume would correspond to a 88 μL injection on a classical 2.1 mm ID column of the same dimensions, resulting in approx. 17.5× overload factor (Aydin et al., 2021). Kafeenah et al. detected estrogens and their metabolites at a level of as low as 60 pM from 200 μL of serum using a 3 μL injection onto a 220 × 0.2 mm column (overload factor 110) via trapping (Kafeenah et al., 2022). Finally, He et al. worked at the overload factor of 50, injecting 3 μL of sample onto a 0.3 mm ID column (He et al., 2022). This tendency to use large overload factors likely arises from the widespread need to achieve the highest concentration of minute quantities of challenging compounds from larger volumes of biological matrix. In addition, these results demonstrate a sufficient loading capacity of both SPP‐ and FPP‐based stationary phases, given the effort undertaken at the sample preparation step to eliminate matrix components.

Another remarkable trend observed across the existing literature is the use of customized ionization sources to achieve μLC/ESI‐MS analysis. Only 30% of the works feature 50 μm ID electrospray needles, the most common and accessible solution to work with lower flow rates. The remaining setups rely on a customized high‐performance solution, either commercially acquired or developed in‐house (Barricklow et al., 2020; Chen et al., 2019; He et al., 2022; Jacquet & Hopfgartner, 2022; Kafeenah et al., 2022; Majewsky et al., 2018; Majuta et al., 2021; Neef et al., 2023; Rochat et al., 2021; Strmeň et al., 2018; Xiao et al., 2021). These findings are further supported by our practical experience demonstrated in Ch. 4 and strongly reinforce the ionization source design as the most crucial contributor to the wider implementation of μLC/ESI‐MS approaches.

Since sensitivity improvement could be considered as the primary driver behind employing miniaturization, a wider occurrence of QqQ systems was expected, especially given their dominance in quantitative studies related to LMWC. However, only 6 of 15 works in this review were performed on such instruments, whereas the majority relied on high‐resolution mass spectrometers (HRMS). This low amount of µLC‐QqQ applications may highlight an additional benefit of having HRMS data for re‐exploration while being able to monitor elusive analytes once only detectable with more sensitive QqQ instruments. Notably, HRMS instruments coupled to μLC provided lower limits of quantitation due to enhanced selectivity (Chen et al., 2019) and demonstrated that quantitation can be achieved with different scan modes, such as full scan (Rochat et al., 2021), sequential window acquisition of all theoretical spectra (SWATH) (Xiao et al., 2021), and alternating all‐ion fragmentation (Chen et al., 2019). Overall, four of the discussed studies (Aydin et al., 2021; He et al., 2022; Kafeenah et al., 2022; Rochat et al., 2021) featured validated protocols, whereas two were performed with >100 samples in a batch (Aydin et al., 2021; He et al., 2022).

Nevertheless, the capability to handle low sample volumes as a distinguishing feature of the miniaturized analytical setups still remains unquestionable, as demonstrated by Barricklow et al. (2020) Chen et al. (2019). Geller et al. exploited this advantage to conduct an exploratory analysis of mouse brain homogenates investigating urea cycle disorder (Geller et al., 2022). Notably, their works also illustrated the feasibility of measuring polar compounds in negative ESI mode using μLC (Geller et al., 2021), although the spray in negative mode tends to be less stable in nano‐LC‐based setups (McClory & Håkansson, 2017). Microflow HILIC can be a potent alternative to the mixed‐mode chromatography for polar compounds. Neef et al. used an in‐house packed BEH amide column, demonstrating an 80‐fold improvement in bile acid analysis compared to conventional systems. However, they also noted nonuniform response to miniaturisation, with S/N ratio being better in only 50% of the analytes (Neef et al., 2023). Finally, the capability of μLC/ESI‐MS to produce high ion current for better fragmentation data was employed in structural studies of the anthelmintic benzimidazole and its metabolites in mice (Majewsky et al., 2018).

Overall, the μLC approach offers an optimal compromise between sensitivity improvement and metabolome coverage in nontargeted studies, as demonstrated by Fitz et al. (2022). Not surprisingly, a rather moderate increase in overall method performance was obtained at the higher range of the microflow region (57 μL/min, 1 mm ID column). However, this increase came practically at no cost, as the microbore column employed and corresponding flow rates are supported by classical binary LC systems. Reducing the flow rate and, consequently, facilitating the management of smaller effluent volumes, is also a potent driver for the advancements of LC and MS technologies. Both emerging (Majuta et al., 2021) and established ionization methods, including atmospheric pressure chemical ionization (Strmeň et al., 2018), exhibit improved performance when operating under microflow conditions. Introducing additional separation dimensions, such as orthogonal two‐dimensional chromatography (Orlandi et al., 2023) or differential ion mobility (DIM) (Jacquet & Hopfgartner, 2022) are accessible to further improve the selectivity of miniaturized setups. Moreover, coupling of μLC with DIM has revealed the influence of eluent on DIM separation process in the analytical flow regimes (Girard et al., 2022).

## 6 CONCLUDING REMARKS

The limited utilisation of μLC approaches in bioanalysis within the existing literature is somehow disheartening, especially considering that advantages of miniaturized systems date back from the 1970s. Recent advancements have propelled analytical instruments towards increased robustness, accuracy and precision, complemented by the availability of precision‐crafted consumables for microanalysis. As practically demonstrated by specific examples, reliable measurements using hyphenated miniaturized chromatography have now become accessible to a wider audience. The increased sensitivity of analytical platforms resulting from these advancements holds the potential to dramatically improve the quantitative results of both targeted and semi‐targeted methods. Moreover, higher ion currents generated by miniaturized systems prove advantageous for emerging alternative fragmentation methods, particularly those suffering from low precursor conversion, such as electron‐activated dissociation (Baba et al., 2021). This opens up new possibilities to uncover a wealth of structural information about metabolites, environmental compounds, and xenobiotics that reside in the lower end of an organism's concentration range. Consequently, this expansion in our analytical capabilities has the potential to deepen our understanding of both biological and environmental processes.

## AUTHOR CONTRIBUTIONS

**Sergey Girel**: Conceptualization; data curation; formal analysis; investigation; writing—original draft. **Isabel Meister**: Conceptualization; investigation; writing—original draft. **Gaetan Glauser**: Conceptualization; resources; writing—review and editing. **Serge Rudaz**: Conceptualization; resources; supervision; writing—review and editing.

## CONFLICT OF INTEREST STATEMENT

The authors declare no conflict of interest.
